# Neoadjuvant pertuzumab plus trastuzumab in combination with chemotherapy for human epidermal growth factor receptor 2 positive breast cancer: a real-world retrospective single-institutional study in China

**DOI:** 10.1186/s12957-024-03365-x

**Published:** 2024-04-06

**Authors:** Dong-Mei Peng, Juan Li, Jia-Xin Qiu, Lin Zhao

**Affiliations:** Department of Breast Surgery, Liaoning Cancer Hospital & Institute, 44 Xiaoheyan Road, NO Shenyang, 110042 P.R. China

**Keywords:** Breast cancer, Human epidermal growth factor receptor 2, Neoadjuvant dual anti-HER2 therapy, Pathologic complete response, Residual tumor

## Abstract

**Introduction:**

Real-world studies on neoadjuvant dual anti-HER2 therapy combined with chemotherapy for breast cancer (BC) are scarce in China. This study aimed to evaluate the efficacy and safety of neoadjuvant dual anti-HER2 therapy combined with chemotherapy in a real-world setting. Moreover, differences in estrogen receptor (ER), progesterone receptor (PR), human epidermal growth factor receptor 2 (HER2), and proliferation cell nuclear antigen (Ki-67) expression pre- and post-neoadjuvant therapy were analyzed.

**Methods:**

Clinical and pathological data of patients with HER2-positive BC who received neoadjuvant dual anti-HER2 therapy combined with chemotherapy at Liaoning Cancer Hospital & Institute, China, between September 2021 and September 2023, were retrospectively reviewed.

**Results:**

Among 179 included patients, a pathologic complete response (pCR) was achieved in 109 patients (60.9%). The univariate analysis results indicated that the hormone receptor (HR) status (*P* = 0.013), HER2 status (*P* = 0.003), and cycles of targeted treatment (*P* = 0.035) were significantly correlated with pCR. Subsequent multivariable analysis showed that HR negative and HER2 status 3 + were independent predictive factors of pCR. Anemia was the most common adverse event (62.0%), and the most common grade 3–4 adverse event was neutropenia (6.1%). The differences in HER2 (34.5%) and Ki-67 (92.7%) expression between core needle biopsy and the residual tumor after neoadjuvant therapy were statistically significant, whereas the differences were insignificant in terms of ER or PR status.

**Conclusions:**

The combination of neoadjuvant trastuzumab and pertuzumab with chemotherapy showed good efficiency, and the toxic side effects were tolerable in patients with BC. In cases where pCR was not achieved after neoadjuvant therapy, downregulation of HER2 and Ki-67 expressions was observed.

## Introduction

Breast cancer (BC) has emerged as the most common cancer worldwide, surpassing lung cancer [1]. Approximately 15% ~ 20% of patients with BC experienced an overexpression of human epidermal growth factor receptor 2 (HER2), which is related to aggressive biological behavior and poor prognosis [2]. HER2-targeted drugs have significantly improved the prognosis of patients with HER2-positive BC [3]. Neoadjuvant therapy (NAT) can effectively reduce the risk of micrometastases and downstage tumor load to increase surgical opportunity [4]. Importantly, clinicians can formulate subsequent treatment strategies based on the efficacy of NAT [5]. Pathologic complete response (pCR) has been demonstrated to be associated with long-term clinical benefits [6, 7], and the pCR rate is the most reliable indicator to predict the efficacy of NAT in BC [4].

The NOAH trial [8] demonstrated that the addition of anti-HER2 treatment to neoadjuvant chemotherapy significantly improved the pCR rate and reduced the risk of recurrence and progression in patients with BC. The NeoSphere trial [9] and its 5-year analysis [6] showed that neoadjuvant chemotherapy combined with trastuzumab and pertuzumab achieved better pCR, progression-free survival (PFS), and disease-free survival (DFS) than trastuzumab plus chemotherapy, thus launching a new era of dual-HER2 blockade for HER2-positive BC. Presently, neoadjuvant dual-target therapy is the preferred treatment for locally advanced HER2-positive BC.

Compared with core needle biopsy, changes of estrogen receptor (ER), progesterone receptor (PR), HER2, and Ki-67 expressions in residual tumor after neoadjuvant HER2-targeted treatment were reported [10–14]. However, there is no consensus on whether testing should be repeated on the residual tumor and the follow-up treatment plan adjusted accordingly.

The use of pertuzumab was limited in China until it was covered by medical insurance in 2019; as a result, there are few real-world studies on neoadjuvant dual anti-HER2 therapy combined with chemotherapy in China [15, 16]. To address this gap, this single-center, retrospective study aimed to evaluate the efficacy and safety of neoadjuvant dual anti-HER2 therapy combined with chemotherapy in a real-world setting, and to examine the pathological characteristics of the residual disease after NAT.

## Methods

### Patients and data collection

This study enrolled patients who received neoadjuvant dual anti-HER2 therapy after being diagnosed with HER2-positive BC by core needle biopsy at Liaoning Cancer Hospital & Institute, China, between September 2021 and September 2023. Their clinical and pathological data, including age, menopausal status, BMI, TNM stage, status of immunohistochemical (IHC) markers (ER, PR, HER2, and Ki-67) in the core needle biopsy and surgical specimen, neoadjuvant regimens, adverse events (AEs) during treatment, the time interval to surgery, type of surgery, and pathological data post-surgery, were collected.

The inclusion criteria were as follows: 1) patients diagnosed with HER2-positive BC by core needle biopsy; 2) patients who received at least four cycles of neoadjuvant dual anti-HER2 therapy; 3) patients with no other treatment performed previously; and 4) patients with an Eastern Cooperative Oncology Group performance status ≤ 1. The exclusion criteria were as follows: 1) the presence of distant metastasis; 2) other concurrent cancers; 3) incomplete clinicopathological data; 4) incomplete surgical treatment; and 5) multifocal BC.

### Neoadjuvant therapy and surgery

The NAT regimens were formulated in accordance with the National Comprehensive Cancer Network (NCCN) and Chinese Society of Clinical Oncology (CSCO) guidelines. Patients underwent blood and biochemical tests before administration of each cycle, and NAT was implemented in the absence of contraindication. Echocardiography was used to assess patients' left ventricular ejection fraction (LVEF) pre- and post-NAT. After completing NAT, the patients were assessed surgically and histopathologically. The surgical plan was based on the clinical tumor size, breast size, and patient preference. The typical surgical procedures included total mastectomy (TM) with axillary lymph node dissection (ALND), TM with sentinel lymph node biopsy (SLNB), breast-conserving surgery (BCS) with SLNB, and breast reconstruction surgery.

### Judgment criteria

Clinical staging was based on the 8th American Joint Committee on Cancer staging. The ER, PR, and HER2 statuses were assessed in accordance with the American Society of Clinical Oncology/College of American Pathologists guidelines (2020). AEs were assessed according to the Common Terminology Criteria for Adverse Events (CTCAE, version 5.0). The Miller–Payne grading system was used to classify the treatment response. Additionally, pCR was defined as no invasive disease in the breast (ypTis/0) and axillary lymph nodes (ypN0) irrespective of ductal carcinoma in situ.

### Statistical analysis

SPSS version 26.0 (IBM, Armonk, NY, USA) was used for data analysis. Univariate and multivariate analyses were performed to analyze the efficacy and influencing factors of NAT. Pre- to post-NAT changes in quantitative biomarkers were tested using the Wilcoxon signed rank test, and qualitative changes were analyzed using chi-square tests. A *P*-value of < 0.05 was considered statistically significant. Graphs were generated using GraphPad Prism 7 (GraphPad Prism Software, Inc.CA, USA).

## Results

### General information and treatments of the patients

**Fig. 1:**
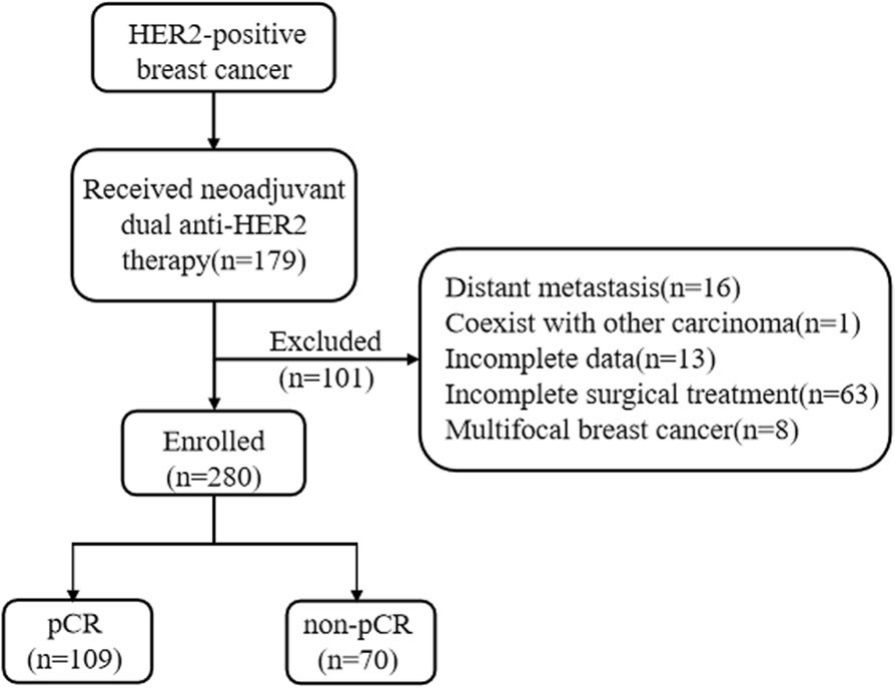
Flow chart of inclusion of the study subjects

Initially, A total of 280 patients were selected,101 patients were excluded after the application of the exclusion criteria, including 16 cases with distant metastasis, 1 with thyroid cancer, 11 with incomplete clinical data, 63 with incomplete surgical treatment (11 cases were lost to follow-up during treatment, 51 had not yet completed treatment at the cut-off date, 1 refused surgery after neoadjuvant treatment), and 8 with multifocal breast cancer. Finally, 179 patients who underwent neoadjuvant dual-target therapy and surgery were enrolled (Fig. 1).

Table 1 shows the clinicopathologic characteristics of the included patients. Notably, the median age of the patients who received neoadjuvant dual-target treatment was 53 (range, 25–75) years, and 57.8% of them were postmenopausal. Among the 179 patients, 97 (54.2%) were HR-positive (defined as ER or PR positive), and the Ki-67 expression in 172 (96.1%) patients was ≥ 20%.

**Table 1:** Characteristics of patients and univariate analysis of factors associated with pCR (*n* = 179)

Characteristics	Total [*n* (%)]	pCR [*n* (%)]	non-pCR [*n* (%)]	*P* value
**Age (years)**				0.974
< 45	32(17.9)	19(59.4)	13(40.6)	
45–59	104(58.1)	64(61.5)	40(38.5)	
≥ 60	43(24.0)	26(60.5)	17(39.5)	
**Menopausal status**				0.770
Pre-menopause	74(41.3)	46(62.2)	28(37.8)	
Post-menopause	105(58.7)	63(60.0)	42(40.0)	
**BMI (kg/m2)**				0.754
< 24	87(48.6)	54(62.1)	33(37.9)	
≥ 24	92(51.4)	55(59.8)	37(40.2)	
**Clinical T stage**				0.110
cT1	13(7.3)	10(76.9)	3(23.1)	
cT2	139(77.7)	87(62.6)	52(37.4)	
cT3	17(9.5)	9(52.9)	8(47.1)	
cT4	10(5.6)	3(30.0)	7(70.0)	
**Clinical N stage**				0.067
cN0	55(30.7)	39(70.9)	16(29.1)	
≥ cN1	124(69.3)	70(56.5)	54(43.5)	
**Clinical stage**				0.320^a^
I	6(3.4)	5(83.3)	1(16.7)	
II	123(68.7)	77(62.6)	46(37.4)	
III	50(27.9)	27(54.0)	23(46.0)	
**HR status**				**0.013**
Negative	82(45.8)	58(70.7)	24(29.3)	
Positive	97(54.2)	51(52.6)	46(47.4)	
**Ki-67**				0.851
< 20%	7(3.9)	5(71.4)	2(28.6)	
≥ 20%	172(96.1)	104(60.5)	68(39.5)	
**HER2 status** (IHC)				**0.003**
2 +	22(12.3)	7(31.8)	15(68.2)	
3 +	157(87.7)	102(65.0)	55(35.0)	
**NAT regimens**				0.181
AC-THP	46(25.7)	23(50.0)	23(50.0)	
TCbHP	124(69.3)	81(65.3)	43(34.7)	
Others ^b^	9(5.0)	5(55.6)	4(44.4)	
**Cycles of targeted treatmen**t				**0.035**
4	53(29.6)	26(49.1)	27(50.9)	
> 4	126(70.4)	83(65.9)	43(34.1)	
**Time to surgery**				0.706
≤ 21 days	66(36.9)	39(59.1)	27(40.9)	
> 21 days	113(63.1)	70(61.9)	43(38.1)	

*Abbreviations: pCR* pathologic complete response, *BMI* Body mass index, *HR* Hormone receptor, *HER2* Human epidermal growth factor receptor 2, *IHC* Immunohistochemistry, *NAT* Neoadjuvant therapy

^a^Fisher Precise inspection

^b^2 patients received THP, 1 received capecitabine combined with THP, 6 from TCbHP changed to THP

Among 130 (72.6%) patients who received carboplatin plus taxane in combination with pertuzumab and trastuzumab (TCbHP), carboplatin was removed from the regimen of six patients due to intolerable AEs. Therefore, only 124 (69.3%) patients completed the TCbHP regimen. Forty-six patients (25.7%) received anthracycline (doxorubicin/doxorubicin) plus cyclophosphamide followed by paclitaxel or docetaxel plus pertuzumab and trastuzumab (AC/EC-THP) every 3 weeks, and nine patients (5%) received other neoadjuvant regimens (2 received THP, 1 received capecitabine combined with THP, and 6 patients from the TCbHP regimen were changed to THP due to AEs).

The time interval from completion of neoadjuvant chemotherapy to surgery was 11–63 days, and the median interval was 24 days (interquartile range, IQR 19–31). Regarding the surgical procedure performed, 143(79.9%) received TM with ALND, 24 (13.4%) underwent TM with SLNB, 8 (4.5%) received BCS with SLNB and 4 (2.2%) received immediate breast reconstruction surgery.

### The efficacy of neoadjuvant dual anti-HER2 therapy and its influencing factors

The results of the postoperative pathological examination showed that the pCR rate was 60.9% (109/179), of which 65.3% (81/124) had received TCbHP and 50% (23/46) received AC/EC-THP. Table 1 summarizes the results of the univariate analysis of pCR-associated factors, in which the patients with HR-negative (pCR: 70.7%, *P* = 0.013), HER2 IHC (3 +) (pCR: 65.0%, *P* = 0.003), and more than four cycles of targeted treatment (pCR: 65.9%, *P* = 0.035) had a high pCR rate. The pCR rate was not significantly associated with the patient’s age (*P* = 0.974), T stage (*P* = 0.110), N stage (*P* = 0.067), clinical stage (*P* = 0.320), or Ki-67 level (*P* = 0.851). Moreover, no statistically significant difference was observed in the pCR of patients who had a ≤ 21 and > 21-day interval of neoadjuvant chemotherapy to surgery.

Twenty-two patients were HER2 IHC (2 +) / FISH-positive. However, only 18 patients had complete records. The median (P25, P75) of HER2/cell number ratio was 8.2 (6.0825–8.8250), the ratio of chromosome 17 centromere /cell number (CEP17/cell) was 2.75 (2.0500–3.9925), and HER2/CEP17 ratio was 2.85 (2.3375–3.8500). Statistical analyses showed that none of the three ratios were associated with the pCR rate of neoadjuvant HER2-targeted treatment (*P* = 0.402, *P* = 0.825, *P* = 0.757).

**Fig. 2:**
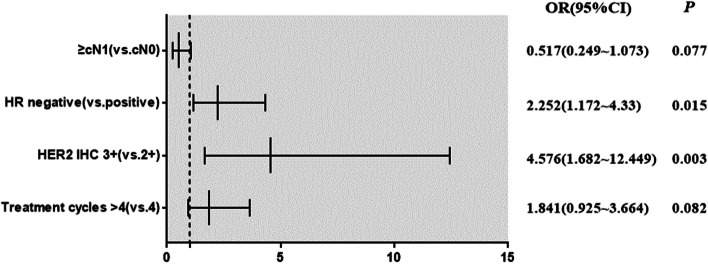
Forest plot of factors associated with pCR

Variables with *P* < 0.1 in the univariate analysis were subjected to the multivariable analysis. Table 2 shows that the HR negative (OR = 2.252, 95% CI: 1.172 ~ 4.323) and HER2 (IHC)3 + (OR = 4.576, 95% CI: 1.682 ~ 12.449) were independent predictive factors of pCR (Fig. 2).

**Table 2:** Multivariate analysis of factors associated with pCR

Parameters	OR (95% CI)	*P* value
Clinical N stage(≥ cN1 vs. cN0)	0.517(0.249 ~ 1.073)	0.077
HR status (negative vs. positive)	2.252(1.172 ~ 4.323)	0.015
HER2 status (3 + vs. 2 +)	4.576(1.682 ~ 12.449)	0.003
Cycles of targeted treatment (> 4 vs. 4)	1.841(0.925 ~ 3.664)	0.066

*Abbreviations*: *HR* Hormone receptor, *HER2* Human epidermal growth factor receptor 2, *OR* Odds ratio, *CI* Confidence interval

### Changes in the ER, PR, HER2, and Ki-67 expression after neoadjuvant HER2-targeted treatment

pCR was not achieved in 70 patients after neoadjuvant dual anti-HER2 therapy. Moreover, only 55 patients had sufficient residual tumor tissue to re-evaluate the expression of the immunohistochemical indicators.

### Quantitative changes of IHC indicators

**Fig. 3:**
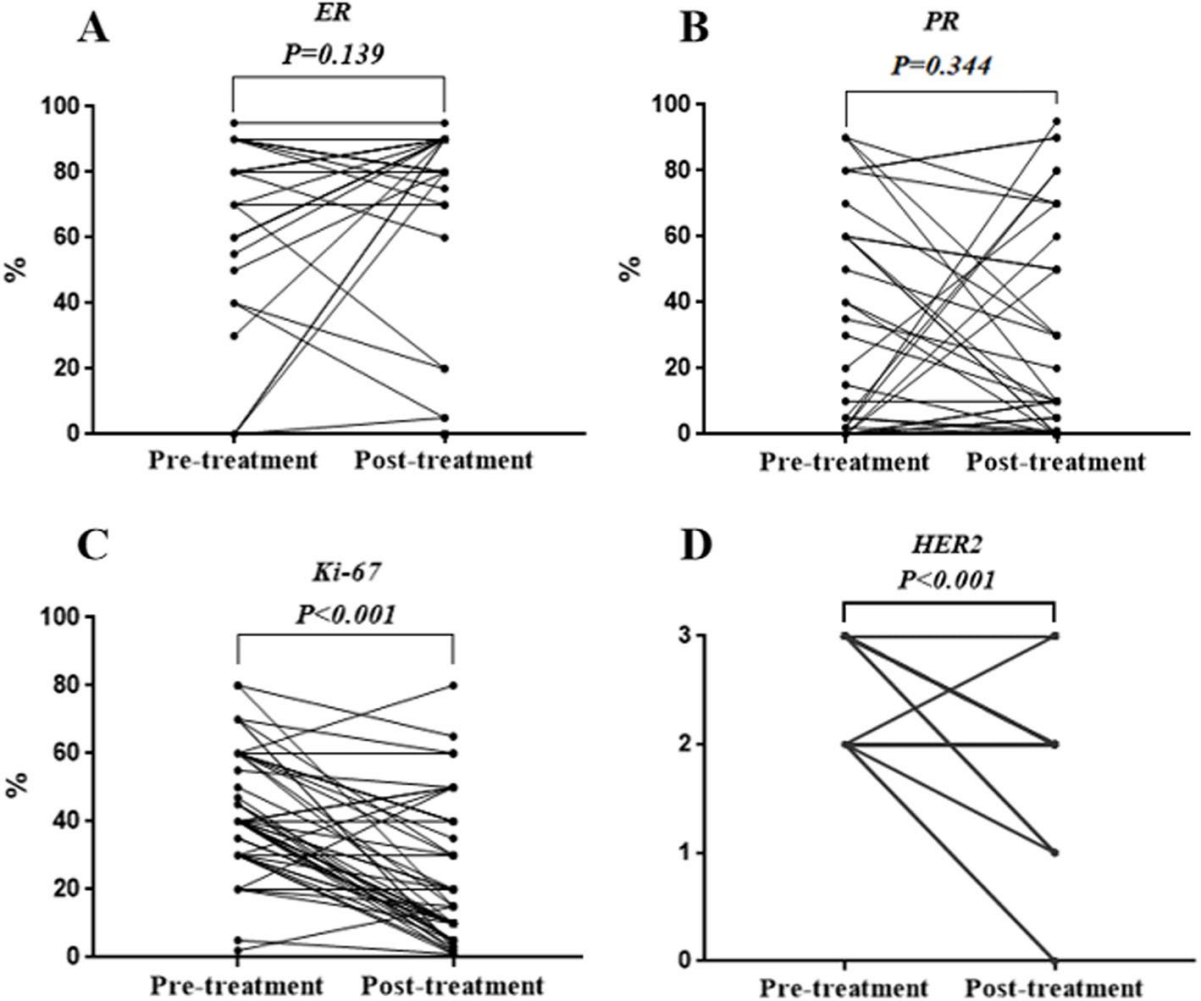
Quantitative changes of IHC indicators. **A** Changes of ER expression. **B** Changes of PR expression. **C** Changes of Ki-67 expression. **D** Changes of HER2 expression

Figure 3 shows a comparison of the quantitative changes in the ER, PR, HER2, and Ki-67 expressions between core needle biopsy and residual lesions. Additionally, 25.4% (14/55) and 16.3% (9/55) of the patients had an increased and decreased ER expression, respectively. The PR expression was upregulated in 16.3% (9/55) and downregulated in 18.1% (10/55) of the patients. Both ER and PR expression changes were not statistically significant (*P* = 0.139, *P* = 0.344) (Fig. 3A, B). More patients had decreased Ki-67 expression post-treatment (81.8%) compared to those with consistent (7.3%) or increased (10.9%) expression (*P* < 0.001) (Fig. 3C). Compared with core needle biopsy results, the HER2 expression of 32.7% (18/55) of the patients changed from 3 + to 2 + ; whereas, 65.5% (36/55) of the patients showed no change (*P* < 0.001) (Fig. 3D).

### Qualitative changes of IHC indicators

**Fig. 4:**
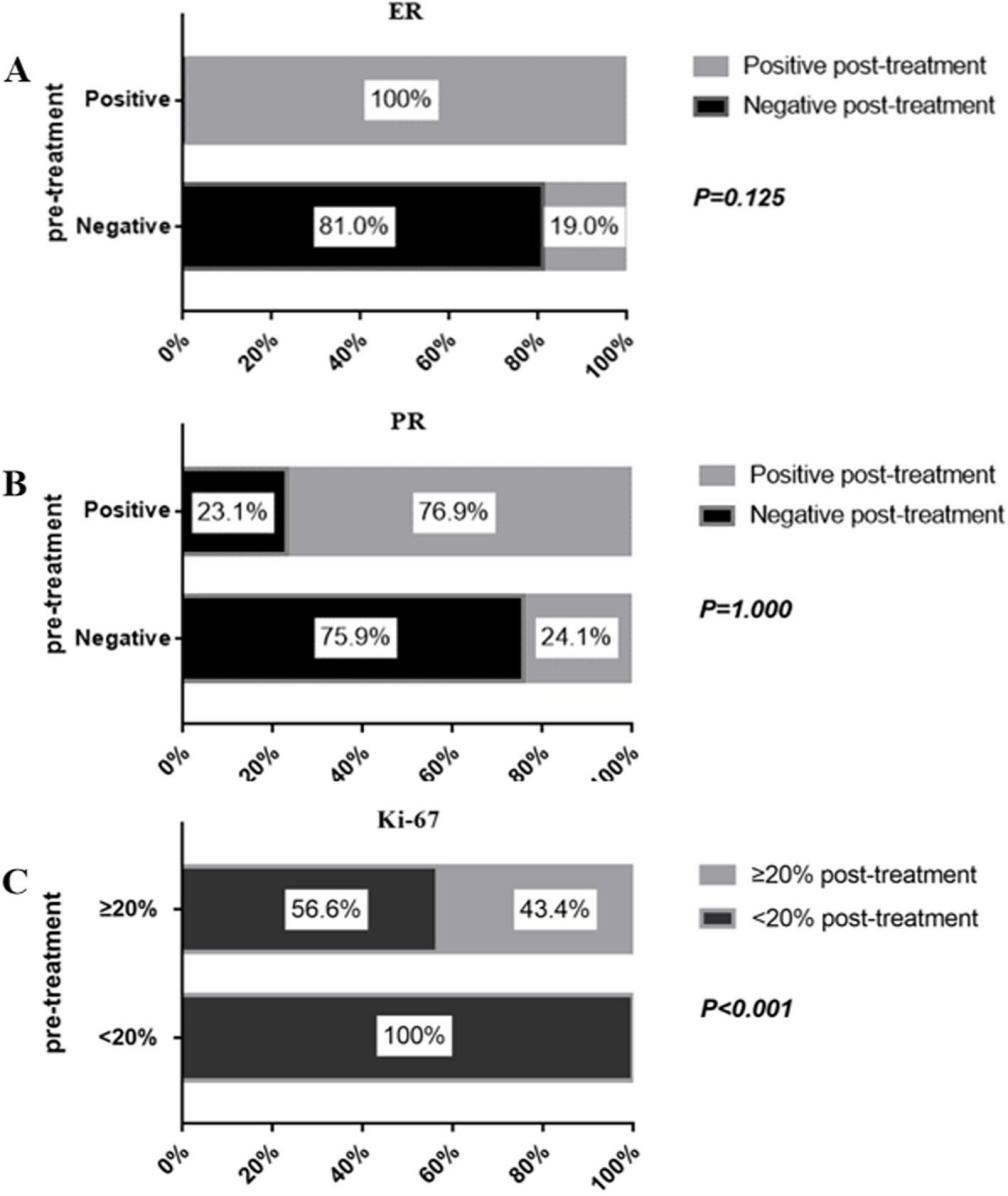
Qualitative changes of IHC indicators. **A** Changes of ER status. **B** Changes of PR status. **C** Changes of Ki-67 levels

Figure 4 shows the qualitative changes in the status of ER, PR, and Ki-67. Following treatment, a difference in ER, PR, and Ki-67 expression was observed in 7.3% (4/55), 23.6% (13/55), and 60% (33/55) cases, respectively. None of the patients with ER-positive at core needle biopsy turned to ER-negative post-treatment. Nevertheless, the ER status of 19.0% (4/21) changed from positive to negative (*P* = 0.125) (Fig. 4A). There were 23.1% (6/26) cases with PR positive-to-negative conversion and 24.1% (7/29) cases with a PR negative-to-positive conversion (*P* = 1.000) (Fig. 4B). Among patients whose Ki-67 expression was ≥ 20% pre-treatment, 56.6% (30/53) had < 20% Ki-67 expression in the residual lesions post-treatment (*P* < 0.001) (Fig. 4C). Four HER2-positive patients became HER2-negative after treatment, and the other 21 patients with HER2 IHC 2 + post-treatment did not undergo FISH testing. Hence, their qualitative analysis of changes in HER2 status was incomplete.

### Safety of neoadjuvant HER2-targeted treatment

Based on the patient’s age, underlying disease tolerance and other aspects, the initial dose was adjusted for some patients. During NAT, 17 (13.1%) patients experienced a carboplatin dose reduction due to AEs among the 130 patients treated with TCbHP, and 6 patients discontinued carboplatin due to intolerable toxicity after reducing the dose (4 patients had bone marrow suppression, 1 had high creatinine, and 1 had severe diarrhea). Among the 46 patients who received AC/EC-THP regimen treatment, only 1 required a docetaxel dose reduction, and no patient had the drugs discontinued.

To ensure the safety of NAT, pegylated granulocyte colony-stimulating factor was prophylactically used for patients with intermediate or high-risk of febrile neutropenia according to the guidelines of CSCO. However, 23.5% of patients still developed leukopenia. The most common AE was anemia (62.0%), and the highest incidence of grade 3–4 AE was neutropenia (6.1%). The common AEs among patients with TCbHP and AC/EC-THP were anemia (69.3% vs. 41.3%), leukopenia (26.9% vs. 13.0%), and neutropenia (23.8% vs. 10.9%). The common grade 3–4 AE was neutropenia (6.9%) in patients who received TCbHP and transaminase increase (4.3%) in those with AC/EC-THP (Table 3).

**Table 3:** Adverse events of neoadjuvant HER2-targeted treatment

Adverse Events (AEs)	Total		TCbHP(*n* = 130)		AC/EC-THP(n = 46)		others(*n* = 3)	
	All grades	Grade3 ~ 4	All grades	Grade3 ~ 4	All grades	Grade3 ~ 4	All grades	Grade3 ~ 4
Leukopenia	42(23.5%)	9(5.0%)	35(26.9%)	4(3.1%)	6(13.0%)	1(2.2%)	1(33.3%)	1(33.3%)
Neutropenia	37(20.7%)	11(6.1%)	31(23.8%)	9(6.9%)	5(10.9%)	1(2.2%)	1(33.3%)	1(33.3%)
Thrombocytopenia	26(14.5%)	6(3.4%)	25(19.2%)	5(3.8%)	0	0	1(33.3%)	0
Anemia	111(62.0%)	4(2.2%)	90(69.3%)	4(3.1%)	19(41.3%)	0	2(66.7%)	0
LEVF decrease	0	0	0	0	0	0	0	0
Transaminase increase	21(11.7%)	4(2.2%)	17(13.1%)	1(0.8%)	3(6.5%)	2(4.3%)	1(33.3%)	0
Creatinine increase	4(2.2%)	1(0.6%)	3(2.3%)	0	1(2.2%)	1(2.2%)	0	0

## Discussion

The pCR rate of patients with HER2-positive BC who received neoadjuvant dual-target combination chemotherapy ranges from 39%~68% [9, 17–22]. There is limited data on neoadjuvant dual-target therapy in China. A domestic study named CSBrS-015 has reported the pCR rate of neoadjuvant dual-target combination chemotherapy to be 57.9% (324/560) [15]. Another multi-center study observed that 46.8% (88/188) of patients with HER2-positive BC achieved pCR [16]. In our study, the pCR rate was 60.9% (109/179), which confirmed the effectiveness of neoadjuvant trastuzumab plus pertuzumab combined with neoadjuvant chemotherapy in treating HER2-positive BC in China.

Existing studies have reported that the pCR rate of NAT in HER2-positive BC was related to the status of HR and HER2 [2, 23–25]. Our results indicate that the status of HR and HER2 were significantly correlated with the pCR rate. Previous clinical trials revealed that patients with HR-negative BC had a higher pCR rate than those with HR-positive BC[18, 19, 21]. The NeoSphere trial reported that 63.2% (36/57) of HR-negative patients with BC achieved pCR, whereas the value was only 26.0% (13/50) for HR-positive patients [9]. This phenomenon may be attributed to the bidirectional crosstalk between ER and HER2 signaling pathways; when HR-positive patients receive anti-HER2 therapy, ER signaling provides cell survival and proliferation stimuli, thereby leading to anti-HER2 resistance [26]. Additionally, compared to patients with IHC (2 +)/FISH-positive, those with HER2 IHC (3 +) had a higher pCR rate, which was consistent with the results of previous studies [27–30]. Some scholars believe that the poor response to anti-HER2 therapy in IHC (2 +)/FISH-positive patients was associated with higher intratumoral heterogeneity [31]. Choi JH et al. [32] and Singer CF et al. [33] found that the pCR rate of neoadjuvant dual anti-HER2 therapy was correlated with the HER2/CEP17 ratio. However, in our study, the pCR rate was not associated with the HER2/CEP17 ratio or HER2 /cell number, which may be attributed to the smaller number of patients who underwent FISH testing (n = 22). Our study also found that patients with more than four targeted treatment cycles had a higher pCR rate. However, Zhou M et al. [16] believed that the number of cycles of targeted treatment was not statistically correlated with the pCR rate.

No consensus was reached on the optimal time interval from completion of NAT to surgery for BC. A retrospective study by Sanford RA et al. [34] reported that a prolonged time interval (> 8 weeks) may affect survival outcomes. Omarini C et al. [35] analyzed a shorter time interval (21 days) and showed that patients who underwent surgery within 21 days after the completion of NAT experienced improvements in OS and DFS. However, Arciero C believed that the time interval has no impact on postoperative complications and prognosis [36]. Presently, clinicians believe that it is appropriate to perform surgery within 2–4 weeks of completing NAT and that it is considered safe within 8 weeks [37]. The time of surgery should be determined according to the clinical situation, and clinicians should seek a balance between recovery from adverse reactions and disease progression control. In our study, 95.5% (171/179) patients underwent surgery within 8 weeks after NAT, but only 36.9% (66/179) patients underwent surgery within 21 days, which may be related to the delayed operation caused by the coronavirus disease 2019 (COVID-19) pandemic.

Patients with residual disease after NAT have a worse prognosis than those who achieved pCR [38]. Hurley J et al. [13] had reported changes in the expression of IHC indicators in residual disease. In the study by Mittendorf EA [12], 20% of patients experienced an ER-negative to ER-positive switch. In another retrospective study, 5.9% of ER-positive patients turned to ER-negative after NAT [39]. A previous study suggested that the upregulated ER expression after anti-HER2 treatment is related to the bidirectional crosstalk between ER and the HER2 signaling pathway [40], and patients with increased ER expression were more likely to develop anti-HER2 resistance [26]. Niikura N et al. [41] reported that the negative conversion rate of PR (18.7%) was significantly higher than its positive conversion rate (9.3%), and the loss of PR expression was related to an improved survival rate. The PR expression changes were not statistically significant in the study performed by Wang RX [39]. The differences in ER and PR expression were not statistically significant in both the quantitative and qualitative analyses in our study. Some studies have shown that Ki-67 expression is significantly reduced after treatment, demonstrating that low Ki-67 levels after neoadjuvant targeted therapy were associated with better prognosis [42, 43].

Interestingly, a decreased expression of HER2 is more common than an increased expression in our study. However, a qualitative analysis of HER2 status changes from positive to negative could not be carried out in this study. A decreased HER2 expression was observed in approximately 8.3–43% of patients with HER2-positive BC after receiving neoadjuvant targeted therapy in previous studies [12–14]. Ignatov T et al. [44] reported that neoadjuvant dual anti-HER2 therapy had a higher HER2-negative conversion rate than single-targeted therapy. Branco FP [11] suggested that HR-negative patients were more likely to have reduced HER2 expression. Wang RX et al. [39] found that patients with a loss of HER2 expression tend to have a higher risk of recurrence.

Presently, there is no definite conclusion on whether the subsequent treatment for BC should be adjusted according to the differences. A further subgroup analysis based on the KATHERINE study showed that adjuvant T-DM1 was beneficial for patients with HER2 absent residual lesions [14]. Giugliano F et al. [45] believed that the bystander effect can partially compensate for the impairment of drug efficacy in the inconsistency of HER2 expression, and the novel antibody–drug conjugates with bystander effect, for example, trastuzumab deruxtecan (T-DXd), may produce more benefits for patients with absent HER2 expression. Since increased ER expression is associated with anti-HER2 resistance, endocrine therapy is recommended for patients whose HR changes from negative to positive after treatment [26].

This retrospective study had incomplete records of subjective AEs such as nausea, vomiting, diarrhea, rash, hair loss, and fatigue. Therefore, only adverse reactions that can be monitored by laboratory tests were emphasized. The widely used granulocyte colony-stimulating factor may exert an impact on our observation of AEs. Anemia was the most common AE in our study, similar to a previous study [23]. No patient experienced a significant decrease in LVEF (LVEF decreased by ≥ 10% from baseline or was between 40–50%), which was consistent with the results of the NeoSphere [9] and TRYPHAENA [46] trials. The balance between efficacy and safety is crucial in clinical settings. For patients with serious AEs, adjusting the treatment dose and strengthening management is necessary. Lv M et al. [30] had proposed that a treatment regimen without carboplatin is recommended for elderly patients with poor tolerance.

This study had some limitations. Firstly, it is a retrospective study without a completion date, making it challenging to evaluate the occurrence and severity of all AEs during NAT. Secondly, this study was conducted during the COVID-19 epidemic, which could have led to delays in the treatment of the patients. The impact of COVID-19 infections on BC development was not considered. Thirdly, the follow-up time was insufficient to analyze long-term survival. Lastly, our study was a single-center study with a limited sample size.

In conclusion, our results show that neoadjuvant dual anti-HER2 therapy combined with chemotherapy has good efficacy, and the toxic side effects are tolerable. The expression of HER2 and Ki-67 in the residual lesions was decreased, and more research on individualized treatment for patients who did not achieve pCR needs to be conducted. We believe that dual anti-HER2 therapy is not the end of HER2-positive BC, but just the beginning.

## Data Availability

No datasets were generated or analysed during the current study.

## Abbreviations

BC: Breast cancer
HER2: Human epidermal growth factor receptor 2
NAT: Neoadjuvant therapy
pCR: Pathologic complete response
PFS: Progression-free survival
DFS: Disease-free survival
ER: Estrogen receptor
RP: Progesterone receptor
Ki67: Proliferation cell nuclear antigen
HR: Hormone receptor
IHC: Immunohistochemistry
FISH: Fluorescence in-situ hybridization
AEs: Adverse events
NCCN: National Comprehensive Cancer Network
CSCO: Chinese Society of Clinical Oncology
LVEF: Left ventricular ejection fraction
TM: Total mastectomy
ALND: Axillary lymph node dissection
SLNB: Sentinel lymph node biopsy
BCS: Breast-conserving surgery
CEP17: Chromosome 17 centromere
